# Recent advances in glucose monitoring utilizing oxidase electrochemical biosensors integrating carbon-based nanomaterials and smart enzyme design

**DOI:** 10.3389/fchem.2025.1591302

**Published:** 2025-04-28

**Authors:** Guan Guoqiang, Qu Liang, Zhao Yani, Wang Pengyun, Kong Fanzhuo, Zhang Yuyang, Lin Zhiyuan, Ni Xing, Zhang Xue, Lu Qiongya, Zou Bin

**Affiliations:** ^1^ School of Food and Biological Engineering, Jiangsu University, Zhenjiang, China; ^2^ School of Food and Bioengineering, Wuhu Institute of Technology, Wuhu, China

**Keywords:** glucose oxidase, electrochemical sensing, carbon nanomaterials, glucose detection, chemical modification

## Abstract

Glucose oxidase (GOx), as a molecular recognition element of glucose biosensors, has high sensitivity and selectivity advantages. As a type of biosensor, the glucose oxidase electrode exhibits advantages such as ease of operation, high sensitivity, and strong specificity, promising broad application prospects in biomedical science, the food industry, and other fields. In recent years, with the advancement of nanotechnology, research efforts to enhance the performance of GOx biosensors have primarily focused on improving the conductive properties and specific surface area of nanomaterials, while neglecting the potential to modify the structure of the core component, GOx itself, to improve biosensor performance. Rapid modification of the GOx surface through chemical modification techniques yields a new modified enzyme (mGOx). Meanwhile, composite techniques involving carbon nanomaterials can be employed to further enhance sensor performance. This article reviews the construction methods and optimization strategies of glucose oxidase electrodes in recent years, along with research progress in their application in electrochemical sensing for glucose detection, and provides an outlook for future developments.

## 1 Necessity of glucose testing

With the rapid development of the social economy, people’s dietary structure has also changed, and carbohydrate-rich diets have become the norm and are increasing day by day. According to the International Diabetes Federation (IDF) in 2019, 463 million people worldwide are currently living with diabetes. China ranks first in the number of diabetic patients, with about 116.4 million people, 25% of the world’s share. The large diabetic community has attracted more and more social attention. People are starting to consciously avoid carbohydrate-rich foods, so foods with low-calorie or non-nutritive sweeteners and sugar-free products have become very popular. According to the GB28050-2011 National Food Safety Standard “General Principles for Nutrition Labeling of Prepackaged Foods”, “sugar-free or sugar-free” specifically refers to a sugar content of no more than 0.5 g per 100 g or 100 mL of solid or liquid food. The complex diversity of food samples makes it necessary to develop a highly sensitive, low-cost, and rapid glucose detection method. To help people understand the sugar content in food and to achieve the purpose of disease prevention (Zhang Y. et al., 2018; Wang et al., 2024). So far, a variety of glucose detection methods have been developed, including fluorescence, optical, sonic, thermal, electrochemical, and colorimetric methods have been used for the detection of glucose due to their simple operation, however, the sensitivity of this method is often poor and insufficient to quantify glucose in the sample, and to circumvent these problems, electrochemical methods have been extensively studied (Li et al., 2021; Ma et al., 2022; Sun et al., 2016). Although different glucose detection techniques have been reported, electrochemical sensors due to their high sensitivity, low cost, and simple instrumentation, it is still considered the most successful analytical tool for glucose detection (Han et al., 2021; Ma et al., 2021; Hu et al., 2024). Glucose electrochemical sensing detection has the advantages of high detection sensitivity, accuracy and rapidity, and low construction cost, which can not only detect human blood glucose levels but also can be used to detect trace amounts of glucose in food, medicine, and biological samples (Bai et al., 2020; Peng et al., 2019), Therefore, electrochemical sensing analysis occupies an important position in glucose detection. Meanwhile, in recent years, the improvement of sensor performance has become a research hotspot, especially in terms of sensitivity, selectivity and long-term stability. For example, Tong et al. (Tong et al., 2024) prepared nanocomposites (PGOx@M-Xene/CS) by efficient electrostatic assembly of GOx polygels (PGOx) onto MXene nanosheets, PGOx can enhance the stability of the enzyme, while MXene’s extensive large specific surface area reduces its influence on enzyme stability. The constructed glucose sensor had a linear range of 0.03–16.5 mM, with a sensitivity of 48.98 μA mM^-1^·cm^-2^, and the limit of detection was 3.1 μM. The current remained at 85.83% of the initial current value after 200 cycles. Ramachandran et al. (Ramachandran et al., 2022) effectively prepared hexagonal CoMn_2_O_4_ electrode material by simple hydrothermal technique using KOH as surfactant and the obtained sensor maintained 85% capacitance after 4,000 consecutive charge/discharge cycles with 81% maximum column efficiency.

## 2 Advances in electrochemical detection sensors for glucose

### 2.1 Glucose oxidase-based electrodes

**FIGURE 1:**
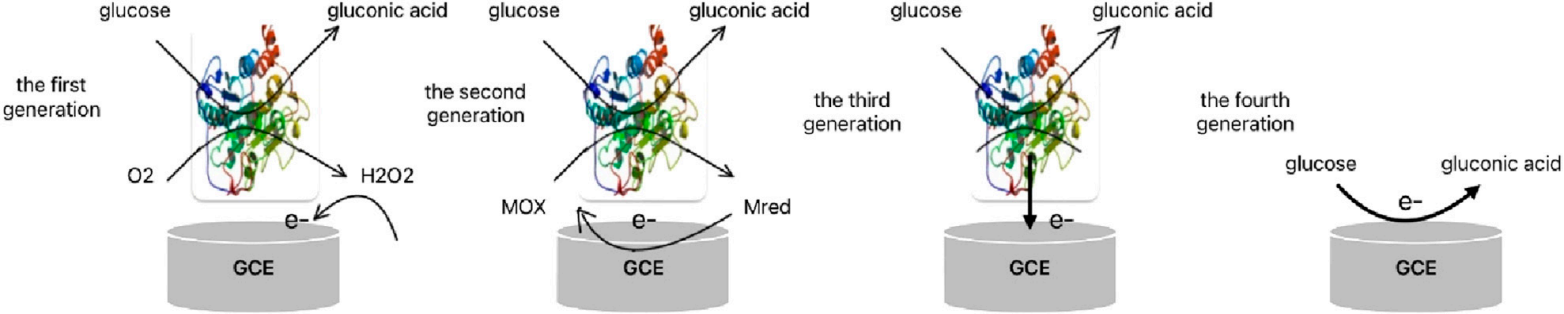
Schematic diagram of electron transfer of fourth-generation electrochemical glucose sensor.

The first enzyme electrodes and enzyme sensors were described by Clark and Updike in the 1960s (Hovancová et al., 2017). In their concept, glucose oxidase (GOx) could be trapped in a semi-dialysis membrane on an oxygen electrode, and glucose concentration could be determined indirectly by monitoring oxygen consumption. Five years later, Updike and Hicks proposed an innovative method for the preparation of glucose analysis enzyme electrodes, which was designed to achieve rapid and accurate determination of glucose concentration in the blood by firmly embedding glucose oxidase (GOx) in a polyacrylamide gel layer overlying an oxygen electrode (Wang P. et al., 2019). Since then, electrochemical glucose sensors have developed rapidly, and electrochemical enzyme electrode technology has become the main method for detecting glucose (Xia et al., 2022). Today, electrochemical glucose sensors have undergone four transformations in principle, forming four generations of sensors, the principle of which is shown in Figure (Figure 1).

#### 2.1.1 First-generation glucose biosensors

The first generation of glucose enzyme electrochemical sensors utilized molecular oxygen as an electron acceptor, and the electrochemical signal was transmitted by measuring the decreasing oxygen concentration or the released H_2_O_2_, but the detection method was greatly affected by dissolved oxygen, and the detection potential was too high, which made the detection results easily affected by other interfering substances (Wen et al., 2020; Bu et al., 2024). On the other hand, the H_2_O_2_ produced during the reaction accumulated, and the concentration increased, resulting in the loss of glucose oxidase activity (Qin et al., 2017).

In the 80s of the 20th century, a large number of studies focused on solving the problems of oxygen interference and redox interference (Zhang et al., 2019). There are many solutions to oxygen interference. Gough et al. (Gough et al., 1985) overcame oxygen interference by increasing oxygen/glucose permeability with the help of a mass migration limiting membrane. Wang et al. addressed oxygen restriction in glucose biosensors by using oxygen-rich Carbon Paste Electrodes (Wang and Lu, 2009). D’Costa et al. avoided oxygen demand deficiency by replacing GOx with glucose dehydrogenase, which does not require oxygen cofactors, and the solution to redox interference was mainly to reduce interference to the electrode surface by selective coating (D Costa et al., 1986). Zhang et al. found that a cellulose acetic-Nafion composite membrane can effectively eliminate electrochemical interference such as acetaminophen (Zhang et al., 1994). Millilista et al. immobilized GOx using an electrochemically synthesized polyphenylenediamine (PPD) membrane that selectively removed ascorbic acid interference (Malitesta et al., 1990; Rong et al., 2019).

#### 2.1.2 Second-generation glucose biosensors

The second-generation biosensor overcame the limitations encountered by the first-generation biosensor. They used redox mediators instead of oxygen to transfer electrons from the enzyme to the surface of the working electrode. The resulting reducing medium is further oxidized on the electrode formation of oxidation mediators, thus the ampere signal was detected. Various organic/inorganic chemicals are used as electronic media, mainly ferrocene derivatives (Monkrathok et al., 2024), ferricyanide (Nikitina et al., 2023), quinones (Mtemeri and Hickey, 2023), transition metal complexes (Wijayanti et al., 2023), and phenothiazine (Teymourian et al., 2020). Campbell et al. realized the detection of glucose by covalently coupling glucose oxidase to a redox medium containing ferrocene, combined with intramolecular electron transfer and electron self-exchange (Campbell et al., 2017). Zhou et al. achieved the detection of glucose by incorporating ferrocene (FC) as an electronic medium and immobilizing glucose oxidase (Gox) on the gate electrode, and tests with simulated blood samples were performed, and the modified sensor showed a bilinear response in the range of 0.6–26.3 mM, whereas conventional sensors (e.g., PEDOT-based) have a narrower linear range (0.5 μM-0.1 mM) (Zhou et al., 2024). Donini et al. (Donini et al., 2020) fixed glucose oxidase to redox graphene and used it for the detection of glucose. Lin et al. (2019) used hydrophilic and positively charged alpha-poly-L-lysine (alpha PLL) as an embedded substrate to fix negatively charged glucose oxidase (GOx) and ferric cyanide (FIC) onto SPCE to construct a disposable second-generation glucose biosensor, and tested in real human serum samples, the sensitivity was significantly improved (from 117.4 to 212.1 nA/mM mm^2^), and the linear range and detection limit were also superior to the pre-improved sensor. The emergence of redox mediators solved the problem of the dependence of glucose sensors on oxygen, but the sensors still had limitations. The presence of redox agents increased the cost of sensors, and many redox agents were biotoxic, limiting the range of sensor applications. Therefore, it is hoped that a way to replace or eliminate redox mediators while maximizing the current intensity of the sensor (Ahmad et al., 2023). Therefore, in the third generation of electrochemical glucose sensors, the biocatalyst was directly bound to the electrode surface where the signal was converted and amplified, thus ensuring that the electron transfer between the biocatalyst and the contact surface of the conductive carrier was as efficient and fast as possible (Khan et al., 1996; Ravikumar et al., 2021).

#### 2.1.3 Third-generation glucose biosensors

**FIGURE 2:**
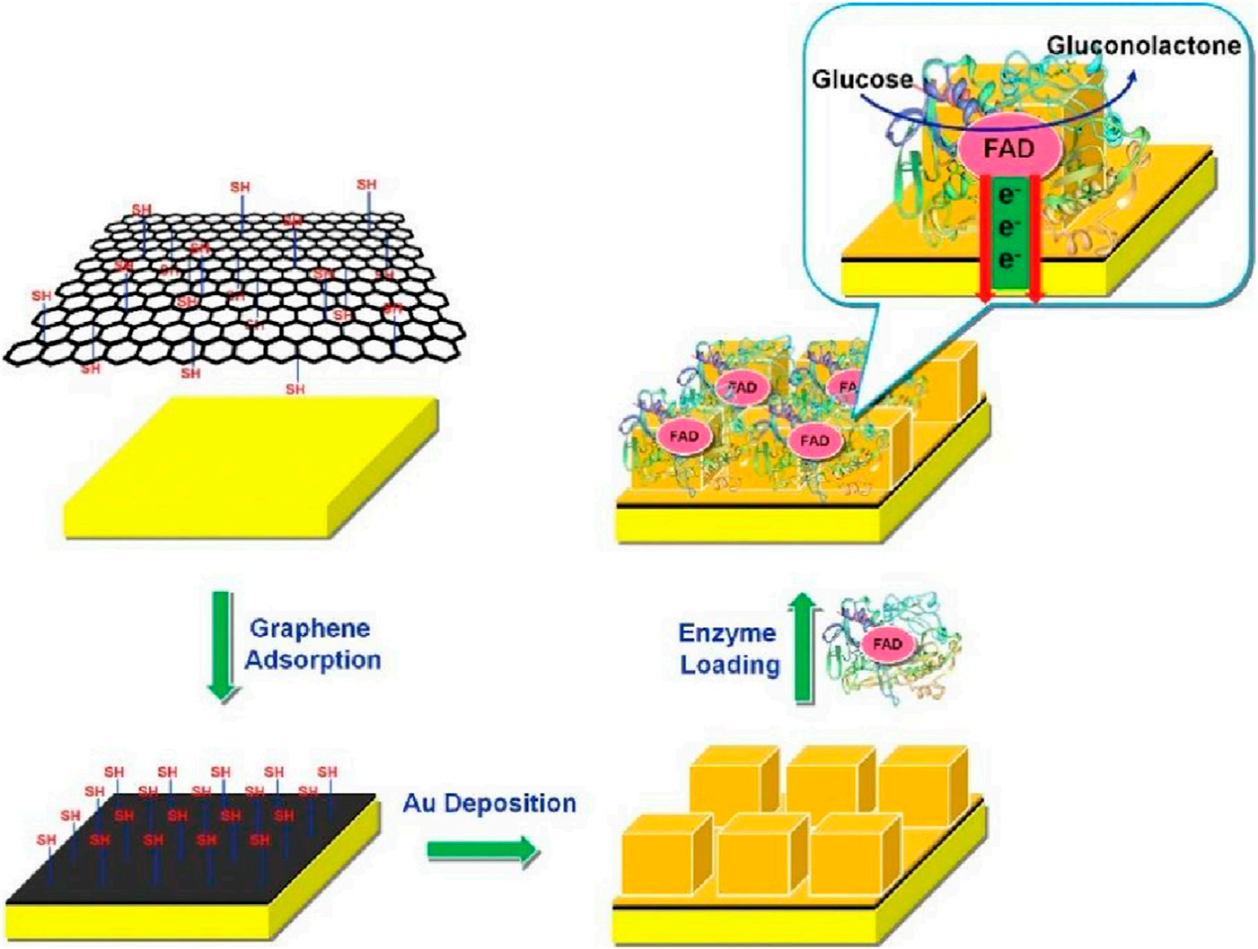
The preparation scheme of Au nanocube/graphene composite film based biosensor. Due to the existence of -SH on graphene surface, oxidase can directly adhere on the film by the interaction produced by the -S-S- from protein and -SH from grapheneReproduced with permission from ref, (Chu et al., 2015). Copyright 2015, Elsevier Publication.

The third-generation glucose biosensor is not dependent on oxygen and redox mediators, and the goal is to transfer electrons directly from the redox site of GOx to the electrode in the absence of a medium. However, the redox center of GOx, flavin adenine dinucleotide (FAD), is deeply embedded in the three-dimensional macrostructure of the enzyme molecule, which acts as an inherent barrier to direct electron transfer between GOx and the electrode. A range of nanomaterials, such as conductive polymers, metal oxides, carbon nanotubes, and graphene, have been used as electrode materials for modified electrodes (Xu et al., 2017; Ma et al., 2019; Shen et al., 2019; Shah et al., 2024). The distance between the active center of the enzyme and the surface of the electrode is effectively reduced to create a suitable environment and promote the smooth progress of the direct electron transfer process between the enzyme and the electrode. Rafighi et al. (2016) synthesized a glucose biosensor using a hybrid of graphene and polyethyleneimine-gold nanoparticles as enzyme carriers, and tested in actual blood samples, the sensitivity of the sensor reached 9.3 μA·mM^-1^cm^-2^, superior to carbon quantum dots reported in the literature (6.1 μA mM^-1^). Tao et al. (Ming et al., 2024; Zhu et al., 2022)modified the sensor by electrochemically depositing gold nanoparticles to enhance its conductivity for better detection of glucose, tested in clinical serum samples, the improved sensor showed only a 13.1% decrease in sensitivity after 7 days in bovine serum compared to a 45.1% decrease in the uncoated version, demonstrating that the PU film significantly extends the service life. Chu et al. (2015) successfully prepared composite carriers by *in situ* electrodeposition of gold nanocubes on a thiol graphene film. These were uniformly distributed on the electrode surface and had a regular nanostructure. This glucose biosensor exhibits high sensitivity and selectivity, and increased sensitivity to 221.0 μA mM^-1^ **·**cm^-2^, 4-7 times higher than similar sensors in the literature, and retained 79.3% sensitivity after 2 weeks of storage (Figure 2). However, the structure of the enzyme protein may change after fixation on the surface of the electrode, which will affect the enzyme activity and the stability of the electrode (Apaliya et al., 2017; Wang L. et al., 2019; Jing et al., 2020). Therefore, preliminary research on third-generation EBGS has focused on how to achieve DET, which mainly involves the following aspects: 1) adding an electron transfer domain to the enzyme itself, 2) modifying with conductive nanomaterials, and 3) modifying with other materials (Song et al., 2024).

Compared with the first and second generations of glucose biosensors, the third generation of glucose biosensors has achieved better results. However, its core principle is still controversial, and whether the DET that generates electrical signals is real is still doubtful. The redox peak may not come from the electron transfer of active enzymes but because the enzyme’s structure is destroyed, resulting in FAD exposure. Moreover, external environmental factors such as temperature, pH, and humidity may affect their dependence on enzyme activity. In addition, the performance of biosensors also depends on the thickness of the enzyme layer, which is high, resulting in signal attenuation or loss (Ou et al., 2021; Yin et al., 2023; Xu et al., 2016).

### 2.2 Glucose dehydrogenase-based electrodes

**FIGURE 3:**
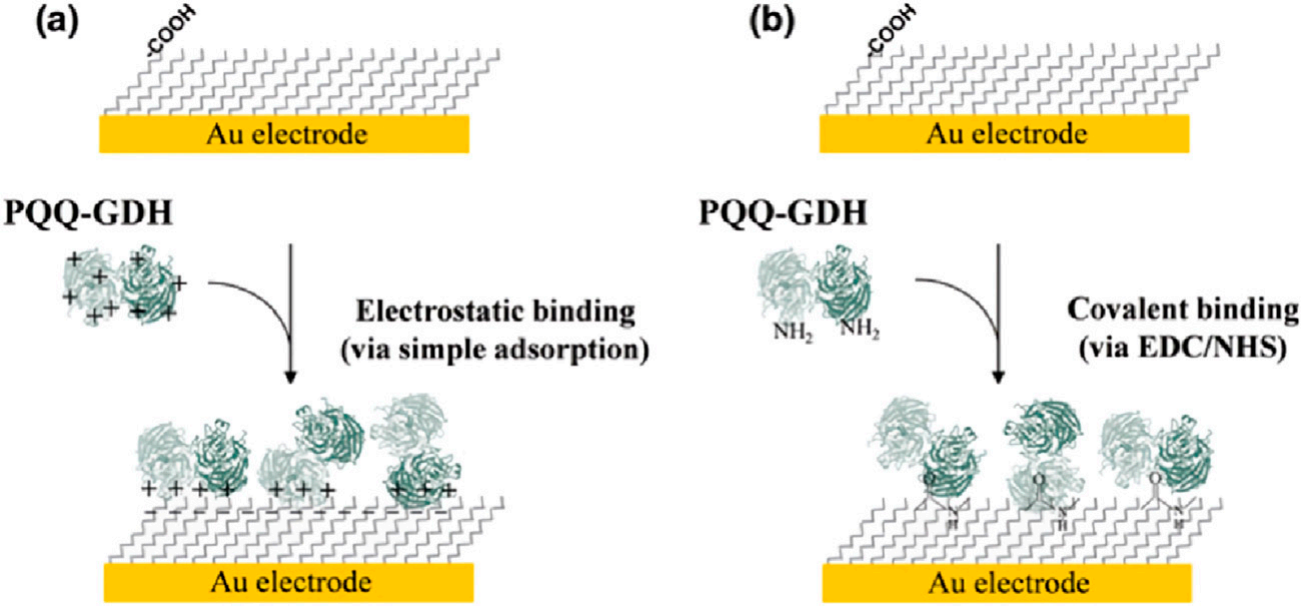
Schematic for binding of PQQ-GDH onto the SAMs of 11-MUA: **(a)** electrostatic and **(b)** covalent binding via EDC/NHS chemistry. Reproduced with permission from ref, (Kim et al., 2012). Copyright 2015, Springer Science Publication.

Glucose dehydrogenase (GDH), an NAD(P)^+^-independent oxidoreductase, has attracted a lot of attention in recent years for its application in glucose sensors. Different types of GDH play an important role in improving the sensitivity, selectivity, and stability of the sensor. Glucose dehydrogenase forms complexes with cofactors such as flavin adenine dinucleotide (FAD), nicotinamide adenine dinucleotide (NAD), or pyrroloquinoline quinone (PQQ) (Zhao et al., 2021). For example, the glucose sensor developed by Kim et al., which involves binding the enzyme to the anionic self-assembled monolayer on the electrode through electrostatic interactions, exhibits high sensitivity. (Kim et al., 2012) (Figure 3). Chen et al. (2022) developed an NAD (+)-dependent dehydrogenase/NPG/SPE biosensing platform for the electrochemical detection of glucose by modifying a screen-printed electrode (SPE) with NPG and NAD (+)-dependent dehydrogenase.

## 3 Non-enzymatic glucose sensor

Non-enzymatic glucose sensors are a technology that does not rely on an enzyme-catalyzed reaction to detect glucose concentration. The core principle of the non-enzymatic glucose sensor lies in the precise detection of glucose concentration through an electrochemical catalytic reaction. The detection system relies on the intrinsic catalytic activity of metal nanomaterials to realize the direct oxidation process of glucose molecules on the electrode surface in an alkaline medium. The catalytic unit is usually constructed with highly active metal nanoparticles, including two systems of noble metals (Au, Ag, Pt) and transition metals (Ni, Co, Cu) (Li H. et al., 2024). These nanostructures significantly enhance the charge transfer efficiency and catalytic site density of the sensor by increasing the effective reaction interface and optimizing the mass transfer channels. Although non-enzymatic sensors circumvent the problem of enzyme activity decay, their selectivity, environmental adaptability and long-term stability are still the main bottlenecks (Table.1). Therefore, glucose oxidase-based sensors are still in the main position for glucose detection.

**TABLE 1:** Comparison of enzyme *versus* non-enzyme sensors.

Category	Core components	Detection mechanism	Sensitivity	Selectivity	Stability	Response time
Enzyme sensors	GOx/GDH	Enzymes catalyze glucose oxidation to produce a measurable electrical signal	High (detection limit as low as μm class)	Excellent (enzyme specifically recognizes glucose)	Low (easy inactivation of enzyme, limited by temperature, pH)	Faster (seconds)
Non-enzyme sensors	Metallic nanomaterials or carbon-based materials	Direct electrochemical catalysis of glucose oxidation, which generates an electric current signal through a reaction on the surface of a metal active site or carbon material	Higer (up to the nM level in some studies, but susceptible to interference in complex samples)	Poor (susceptible to interference from electroactive substances such as ascorbic acid, uric acid, etc., requires additional functionalization)	High (no biocomponents, resistant to high temperatures, wide pH range)	Faster (milliseconds as no enzyme catalytic step required)

The fourth-generation glucose biosensor is a non-enzymatic glucose sensor that uses an artificial substance with enzyme-like catalytic properties to replace glucose oxidase and oxidize glucose directly on the surface of the electrode. Fourth-generation glucose sensors (FGGS) are designed to enhance glucose-sensing technology and reduce the number of intermediate stages equired for glucose measurement. Diagnostic efficiency and cost-effectiveness can be improved by using these sensors, which are fabricated using electrocatalytic copper nanostructures (Naikoo et al., 2022; Kilic et al., 2023). Ahmad et al. fabricated an electrochemical-based non-enzymatic glucose biosensor using engineered layered CuO nanoleaves, which shows high sensitivity (1467.32 *μ*A/(mM cm^2^)), linear range (0.005–5.89 mM), and detection limit of 12 nM (S/N = 3) (Ahmad et al., 2021). The fourth generation has the advantage of using chemically derived materials, which is more suitable for large-scale production, and the chemically produced identification parts can exert better uniformity and reproducibility compared to enzymes prepared by biotechnology. On the other hand, this type of device can be affected (Pohanka, 2021). Therefore, the use of glucose oxidase electric sensors for glucose detection is still the mainstream.

## 4 Enhancement of glucose oxidase activity stability

**FIGURE 4:**
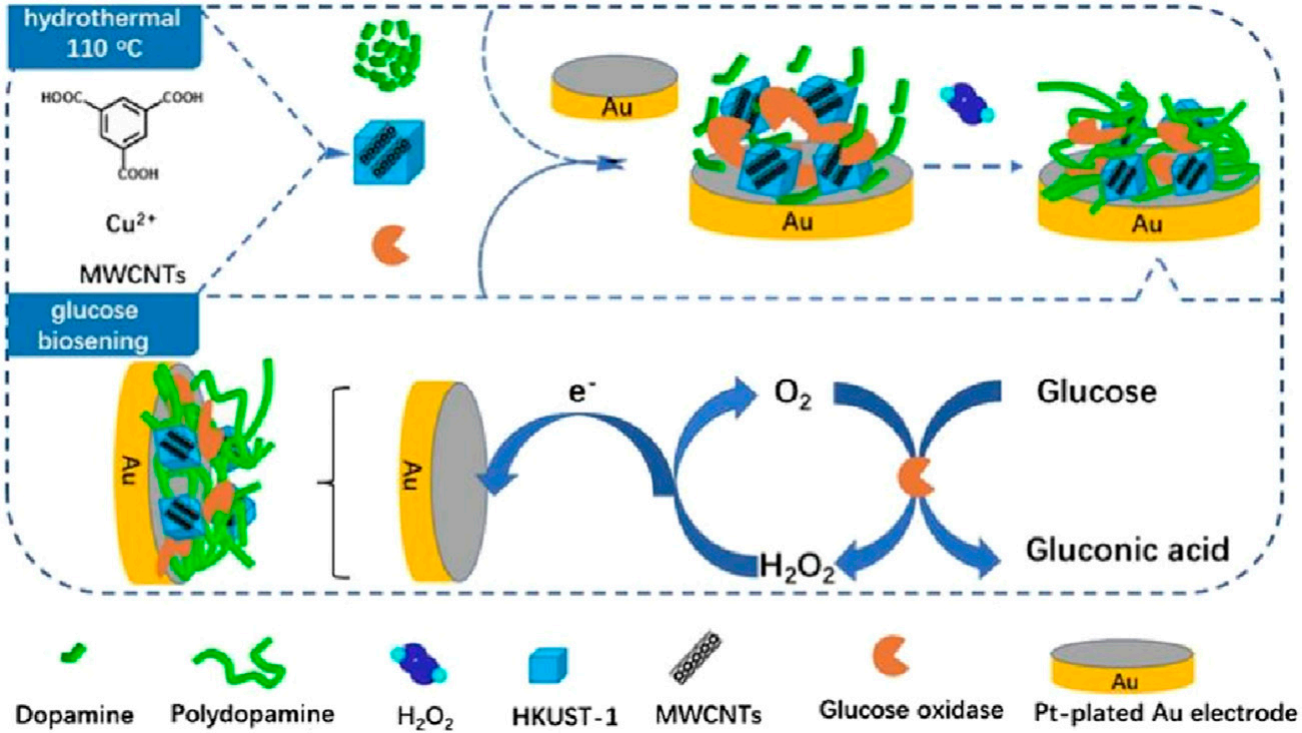
Copper-based metal–organic frameworks (MOF) and multi-walled carbon nanotubes (HKUST-1-MWCNTs) composite were synthesized by one-step hydrothermal method, and PDA-enzyme-HKUST-1-MWCNTs composite was prepared by one-pot method for the construction of glucose biosensors. Reproduced with permission from ref, (Chen et al., 2023). Copyright 2022, Springer Science Publication.

In the development of glucose biosensors, the enhancement of glucose oxidase activity stability is the core technical difficulty. In recent years, the linkage strategies for Gox activity attenuation have focused on the following directions: nanomaterial encapsulation protection: encapsulating GOx by metal-organic frameworks (MOFs) or mesoporous silica to limit the enzyme molecular conformational changes. For example, Mao et al. (Mao et al., 2025) successfully synthesized glucose oxidase (GOx)@Zn-HHTP, which significantly improved the stability of the encapsulated GOx and was applied to construct an ECL glucose sensor with 8.5-fold increase in sensitivity and 25-fold decrease in detection limit, which was successfully applied to detect glucose in sweat. Covalent cross-linking enhancement: Glutaraldehyde or genetically engineered bifunctional cross-linkers (e.g., SpyCatcher/SpyTag) were used to enhance the enzyme-electrode interface binding. For example, Chmayssem et al. (2023) used a chitosan-based hydrogel to capture glucose oxidase (GOx) and crosslinked the entire substrate with glutaraldehyde, and the resulting biosensor was able to maintain its stability over 6 months of storage. Biomimetic polymer coatings: polydopamine (PDA) or amphoteric ionic polymers (e.g., poly (sulfobetaine)) were utilized to form antifouling coatings. For example, Chen et al. (2023) combined the good hydrophilicity and biocompatibility of PDA with the high loading properties and peroxidase-like activity of HKUST-1 to synthesize the PDA/HKUST-1/MWCNTs/GOx biosensor, which was used for glucose detection with a sensitivity of 178 μA mM^-1^cm^-2^, a linear range of 0.005 mM, and a limit of detection of 0.12 μ M. The initial current response value remained at 82.0% after 30 days (Figure 4).

## 5 Chemical modification of glucose oxidase

### 5.1 Introduction to glucose oxidase

In the presence of molecular oxygen, glucose oxidase catalyzes the oxidation of β-D-glucose to D-glucono-delta-lactone and hydrogen peroxide. The resulting D-glucono-delta-lactone is sequentially hydrolyzed to D-gluconate by lactonase, and the resulting hydrogen peroxide is hydrolyzed by catalase to oxygen and water (Bauer et al., 2022). GOx has a relative molecular mass of 130 × 10^3^–175 × 10^3^ and exists as a glycosylated homodimer, with each subunit noncovalently bound to one flavin adenine dinucleotide (FAD) molecule, and the two subunits are bound to each other by forces such as salt bridges and hydrogen bonds (Huang et al., 2022). In the catalytic process, GOx uses molecular oxygen as an electron acceptor and FAD coenzyme as an electron carrier to catalyze specifically the formation of D-glucose-delta-lactone and hydrogen peroxide (H_2_O_2_) from β-D-glucose (Wang et al., 2022). GOx is most active under weakly acidic conditions, and the pH range in which activity is stable is 3.5–7.5; other than that too much acid and too much alkali will inactivate the enzyme molecule. GOx is available from a variety of sources. Bacterial, fungal, herbal, and animal sources are the main ones. Fungi are considered to be the richest source and are widely used for industrial applications, *A. niger*, and P. glaucoma were the first identified sources of GOx isolates, and glucose oxidase isolated from Aspergillus niger is considered to be the most stable (Khatami et al., 2022; He et al., 2022; Guo et al., 2021; Shang et al., 2019). Glucose oxidase has been widely used in biomedical applications (Min et al., 2023), bio-Fenton oxidation (Vaidyanathan et al., 2023), feed field (Liang et al., 2023), textile bleaching (Tzanov et al., 2002), reducing wine processing (Pickering et al., 1998), food packaging deaerator (Ge et al., 2012), etc.

### 5.2 Methods of chemical modification of enzymes

Chemical protein modification provides a large toolbox for the study and modification of enzymes, which play key roles in many important biological events in organisms; in particular, by associating desired properties/functions (affinity probes, fluorophores, reactive tags, etc.) with naturally or synthetically modified amino acid residues, chemical protein modification provides a useful way to identify enzyme locations and elucidate enzyme functions (Yu et al., 2022; He et al., 2018; Yan et al., 2024).

Chemical modifications of enzymes can be divided into covalent modifications, non-covalent modifications, targeted modifications, and macromolecular coupling (Table.2). The specific modification mechanisms are shown in the following table (Table.3). Surface modification is carried out using functionalized small molecules bearing alcohol, aldehyde, carboxylic acid, or isothiocyanate groups, and these reactions are carried out on the enzyme surface with exposed functional groups to form covalent bonds (Giri et al., 2021; Noro et al., 2022). Polymer coupling is the most common strategy for macromolecular modification enzymes. Among macromolecules such as polyglycol, polypropylene, and dextran, polydiethanolization is the most studied method, such as covalent polyglycol bonding with partial molecules or macromolecules (Noro et al., 2022; Li T. et al., 2024; Zhang et al., 2020; Chen et al., 2024). Chemical modifications of enzymes can alter affinity, specificity, or stability, while selective modifications enable the labeling of enzymes, allowing insight into complex biological processes, significant improvements have been made in the field of chemical modification/capture strategies for proteomic analysis, these methods emerged to be able to analyze the activity of enzymes in the body, and the chemicals used are often site-specific variants of those chemicals that are more commonly used to modify proteins and enzymes (Diaz-Rodriguez and Davis, 2011; Ren et al., 2018).

**TABLE 2:** Classification of chemical modifications of enzymes.

Modification type	Mechanism of action	Technical examples	Impact on GOx performance
Covalent modification	Introduction of functional groups or molecules on the GOx surface through chemical bonding (e.g., amide bonds, thioether bonds)	Amino and carboxyl cross-linking (e.g., Ferrocene-GOx complex), acylation of lysine residues (citric anhydride modification)	Enhanced electron transfer efficiency, improved thermal stability, expanded pH tolerance range
Noncovalent modification	Functional materials based on physical adsorption or electrostatic loading	Porous silicate-loaded Gox, graphene/polyaniline complex embedding	Maintains enzyme activity, improves immobilization efficiency, reduces conformational changes
Directional modifier	Specific modifications targeting the GOx active center (FAD cofactor)	Epoxy acid modification of FAD with polyethyleneimine coupling and coenzyme analog replacement	Shortening of electron transfer pathways, enhancement of catalytic efficiency and coenzyme stability
Macromolecular coupling	Covalent binding of GOx via polymers (e.g., PEG, dextran)	PEGylation modification to improve enzyme solubility and dextran modification to enhance organic solvent tolerance	Extends enzyme life, improves biocompatibility, reduces non-specific adsorption

**TABLE 3:** Mechanistic explanation of chemical modification of enzymes.

Modification type	Mechanism explanation	
Covalent modification	Hydrophobic modification: forming a rigid hydrophobic core, reducing conformational fluctuations caused by water molecule intrusion, and improving thermal stability	Hydrophilic modification: Enhance the dispersion of the enzyme in the aqueous phase to reduce aggregation inactivation
Noncovalent modification	Conductive network enhancement: Utilizing the high conductivity of carbon nanomaterials (e.g., graphene, carbon nanotubes) to construct a continuous electron transfer pathway and reduce interfacial resistance	Micropores (<2 nm) in porous materials (e.g., hollow carbon spheres) limit the conformational changes of the enzyme molecule and reduce the inactivation caused by thermal movement
Directional modifier	Direct electron transfer by anchoring the electron mediator to the vicinity of the FAD coenzyme through chemical modification (e.g., epoxy acid coupling) or genetic engineering (e.g., introduction of cysteine tags)	Introduction of hydrophilic groups (e.g., carboxymethyl) at the substrate entrance to lower the substrate binding energy barrier and enhance catalytic efficiency
Macromolecular coupling	PEGylation modification reduces hydrophobic interactions between enzyme molecules through spatial site resistance	Dextran coupling forms a hydrophilic protective layer, preventing protease from approaching the enzyme molecule, extending the lifetime from 1 week to 6 months

### 5.3 Research progress on glucose oxidase modification

**FIGURE 5:**
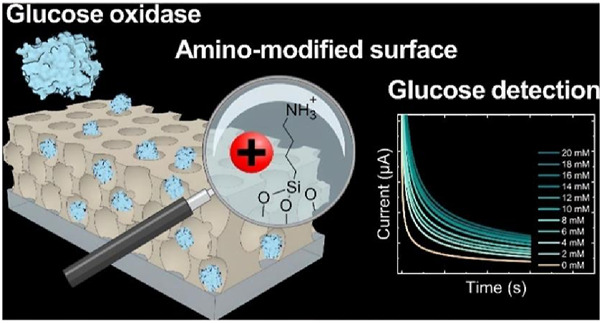
Poly (isoprene)-block-poly (ethylene oxide) (PI-b-PEO) micelles were co-assembled with aluminum silicate nanoparticles in solution and then spin-coated onto the working electrode to form a thin film. The hybrid coating was then calcined to condense the inorganic nanoparticles into a continuous matrix and remove the block copolymer (BCP) micelles, resulting in pores. Reproduced with permission from ref, (Fornerod et al., 2023). Copyright 2023, ACS Publication.

The specific recognition of glucose by glucose oxidase makes glucose oxidase biosensors the most common electrochemical biosensors for glucose detection. However, the current sources and types of glucose oxidase are limited, and rapid molecular modification of the enzyme, such as chemical modification techniques, is needed to alter its activity and selectivity. In recent years, the surface modification of glucose oxidase has attracted the attention of researchers (Table.4) (Liu et al., 2024). Back in 1991, Kunugi et al. (1992) introduced a ferrocenyl group on glucose oxidase by combining the carboxyl group on ferrocene acid with the amino group on glucose oxidase to form a modified enzyme, the modified enzyme can be detached from oxygen for electron transfer with the electrode. Halalipour et al. (2020) attached phenyl derivatives to the carboxyl side chain or amino side chain of glucose oxidase (Gox) and modified Gox with hydrophobic aniline and benzoate, respectively, which showed that aniline-modified Gox had the highest catalytic efficiency, followed by benzoate-modified Gox, and the natural Gox performed the worst. It is demonstrated that hydrophobic modification increases Gox activity and is more resistant to high temperatures, at 80°C and 240 MPa, the rate of inactivation of aniline-modified GOx was 3.7-fold lower than that of the natural enzyme and 2.8-fold lower than that of the benzoic acid-modified enzyme. Hosseinkhani et al. (Hosseinkhani et al., 2004; Shi et al., 2019; Jiang et al., 2020) used citric anhydride to modify the lysine residues of glucose oxidase chemically, the pH tolerance of the modified enzyme was enhanced, and UV and fluorescence spectra also indicated that the chemical modification resulted in more exposure to hydrophilic residues. In addition, Liu et al. (Liu et al., 2013) also utilized the carboxyl group on 1-pyrenebutyric acid to covalently bind to the amino group on the surface of glucose oxidase, so that the enzyme surface carries a pyrenyl group with a conjugated structure, which was then loaded on graphene and assembled into a glucose oxidase electrode, which greatly improved the detection range of glucose concentration, the linear detection range of the modified sensor was 0.2–40 mM with a detection limit of 0.154 mM (S/N = 3), which greatly improved the detection range of glucose concentration, and was tested in real human serum samples, which greatly improved the detection range of glucose concentration, with a 5.1% decrease in activity in the first week, and still retaining 82.2% activity after 4 weeks. In addition to the surface modifications of the Gox, Zappelli et al. (Zappelli et al., 1978; Hou et al., 2017) modified FAD with epoxy acid and subsequently coupled it with polyethyleneimine to form a macromolecular FAD, which showed a 12-fold increase in stability compared to unmodified FAD in a circulating system with alanine as substrate. Fornerod et al (2023) used porous aluminosilicates and silicates for surface modification of Gox loading, the glucose detection sensitivity of the amino-modified electrode was 0.26 μA/mM (0–14 mM), which was higher than that of the unmodified electrode of 0.16 μA/mM (0–8 mM), and the detection limit of the modified electrode was 1.4 mM lower than that of the unmodified electrode (3.6 mM) (Figure 5). Lv et al. (2021) modified Gox with amines to obtain enzymes with higher enzymatic activity and to give the sensor a higher peak current intensity, the electron transfer rate constant of the amine-modified sensor, 2.54 s^-1^, was 1.39 times higher than that of the unmodified sensor (1.83 s^-1^). It can be seen that chemical modification can quickly and inexpensively change the properties of glucose oxidase, and glucose oxidase will be more and more widely used.

**TABLE 4:** Chemical modification of glucose oxidase.

Modification type	Functionalization strategy	Performance enhancement	Application scenarios	References
Covalent modification	Ferrocen covalently binds to the amino group on the surface of Gox to form an electron-mediated complex	Electron transfer efficiency increased by 2.5-fold, oxygen dependence reduced by 80%	Medical diagnostics (blood glucose monitoring)	Kunugi et al. (1992)
Covalent modification	Aniline hydrophobically modifies Gox carboxyl/amino group to form a rigid hydrophobic core	Catalytic efficiency increased to 1.8 times that of natural enzyme, activity retention increased from 40% to 85% at 70°C	Food testing (high-fat samples)	Halalipour et al. (2020)
Covalent modification	Modification of Gox lysine residue by citric anhydride to enhance hydrophilicity	Expanded pH tolerance from 4.0-7.5 to 3.0-9.0 and increased activity retention from 40% to 90% in acidic environment	Industrial catalysis (bioreactors)	Hosseinkhani et al. (2004)
Covalent modification	Pyrene moiety covalently bound to Gox surface via carboxyl-amino group and complexed with graphene	Linear range of detection extended from 0.1 to 10 mM to 0.01–50 mM, sensitivity up to 98.7 μA mM^-1^·cm^-2^	Highly sensitive biosensing	Liu et al. (2013)
Directed modification	FAD coenzyme modified by epoxy acid coupled with polyethyleneimine to form stable coenzyme-polymer complexes	12-fold increase in coenzyme half-life and 3.5-fold increase in current response strength	Long-term stability sensor	Zappelli et al. (1978)
Non-covalent modification	Aminoated porous silicate loaded with Gox, immobilization of enzyme molecules by electrostatic adsorption	Aminoated porous silicate loaded with Gox, immobilization of enzyme molecules by electrostatic adsorption	Complex sample detection (blood)	Fornerod et al. (2023)
Macromolecular coupling	PEGylated modification of Gox surface amino groups to form a hydrophilic protective layer	Enzyme activity retention in organic solvents increased from 30% to 75%, extending the lifetime to 6 months	Industrial enzyme-catalyzed reactions	Kajiwara et al. (2019)

## 6 Multifunctional applications of carbon nanomaterials for glucose sensing

Since the birth of the first glucose oxidase electrode in 1962, people have been trying to find suitable materials to modify glucose oxidase on the electrode surface and maximize enzyme activity. Carbon nanomaterials have attracted great research interest due to their unique size, strength, electrical and surface area properties, and good biocompatibility and stability (Zeng et al., 2016). Therefore, carbon nanomaterials have become popular materials in the field of catalysis and sensing (Table 5). Currently people apply carbon nanomaterials to electrochemical sensors, which not only simplifies the size of the sensor, but improves the sensitivity as well as the stability of the electrode, but also greatly shortens the response time so that the electrochemical sensor is toward the direction of practical, miniaturization, and multifunctional development (Table 6) (Kaçar et al., 2014; Bi et al., 2018; Dong et al., 2024).

**TABLE 5:** Classification of carbon nanomaterials in glucose sensors.

Material classes	Structural properties	Functional advantages	Typical study cases
One-dimensional materials			
Carbon nanotubes (CNTs)	Tubular structure、sp^2^ hybridization、high L/D ratio (L/D > 10^6^)	High electrical conductivity (10^4^ S/cm)、strong adsorption capacity、 large specific surface area (∼500 m^2^/g)	(Hyun et al., 2015): CNTs immobilized GOx, sensitivity 53.5 μA mM^-1·cm -2^ with 86% retention of 2-week activity
Carbon nanofibers (CNFs)	Fibrous porous structure、 50–200 nm diameter	High mechanical strength (∼3 GPa)、 3D interconnected pores (pore sizes 2–50 nm)	(Zhang et al., 2018b): detection limits as low as 0.015 mM, recoveries in the range of 101.0%–104.8%
Two-dimensional materials			
Graphene (GR)	Single atomic layer honeycomb structure, theoretical specific surface area 2,630 m^2^/g	Ultra-high carrier mobility (2 × 10^5^ cm^2^/V·s), abundant edge active sites	(Wu et al., 2022): Pt/GO composite electrode sensitivity 11.64 mA mM^-1^, response time <3 s
Graphene oxide (GO)	Layered structure modified with oxygen-containing functional groups	Good hydrophilicity, easy functionalization (e.g., -NH_2_, -COOH modification)	(Pakapongpan and Poo-Arporn, 2017): Fe_3_O_4_/GO self-assembled system, detection limit 0.1 μM
Graphyne (GDY)	sp-sp^2^ hybridized network, intrinsic pores (∼0.5 nm)	Excellent porosity, high density of catalytically active sites (∼10^15^ sites/cm^2^)	(Liu et al., 2019): Fe-GDY/GOx electrodes in the range of 5–160 μM glucose concentration, R^2^ = 0.998
three-dimensional material			
Carbon aerogel (CA)	Three-dimensional porous network, density 0.1–0.5 g/cm^3^	Ultra-low density, high electrical conductivity (∼10 S/cm), compression resilience >90%	(Yu et al., 2015): ZrP-CA/GOx Linear calibration in the range of 0.12–2.0 mM, sensitivity 5.56 μA mM^-1·^cm^-2^
zero-dimensional material			
Carbon quantum dots (CQD)	Particle size <10 nm, surface rich in -OH/-COOH groups	Tunable fluorescence properties, excellent biocompatibility, easy surface functionalization	(Hu et al., 2020): CdTe QDs/CQDs Concentration detection range from 0 mM to 13 mM, detection limit 0.223 mM
Nanodiamonds (NCD)	Diamond cores (∼5 nm) + surface sp^2^ carbon layer	Chemically inert, high hardness, surface functionalization (e.g., -NH_2_ modification)	(Zhao et al., 2006): N-NCD/Gox has a wide linear calibration range of 10 μM–15 mM and a low detection limit of 5 μM

**TABLE 6:** Mechanism of the effect of different carbon nanomaterials on sensor performance.

Material classes	Long-term stability	Anti-interference performance
One-dimensional materials		
Carbon nanotubes (CNTs)	Literature sensor retains 86% of initial activity after 14 days of continuous use in a simulated serum environment (25°C, pH = 7.4)	π-π conjugate shielding: the sp^2^ hybridized surface of single-walled carbon nanotubes forms a π-π stacking with the tryptophan residues of GOx, which preferentially adsorbs glucose molecules (hydrophobicity), while repelling hydrophilic interferences such as uric acid, ascorbic acid, and others
Carbon nanofibers (CNFs)	The sensor in the literature retained 71.9% activity after 30 days	3D mesh filtration: sub-micron pores formed by interwoven fibers block macromolecular interferents through size exclusion effect, while allowing glucose to diffuse freely
Two-dimensional materials		
Graphene (GR)	The sensor in the literature showed only a 5% decrease in sensitivity after 30 repetitions in tomato juice, indicating excellent short-term stability	Surface charge repulsion: negatively charged surfaces inhibit negatively charged interferents through electrostatic repulsion
Graphene oxide (GO)	The current response of the sensor in the literature remained 95.6% of the initial value after 1 month of storage at 4°C	Surface charge repulsion and negatively charged surfaces suppress negatively charged interferences through electrostatic repulsion
Graphyne (GDY)	High short-term stability of the literature sensor	Catalytic site specificity, graphyne modification site preferentially catalyzes glucose oxidation
Three-dimensional material		
Carbon aerogel (CA)	After 90 scans of the sensor in the literature, the peak current decreased by less than 8%, indicating good stability	Hierarchical pore design: macroporous-mesoporous-microporous three-stage structure (pore size distribution of 50 μm-2 nm) enables the mass transfer rate of interferents (e.g., ascorbic acid diffusion coefficient of 1.2 × 10^−5^ cm^2^/s) to be only 1/3 of that of glucose by the difference in the diffusion paths. only 1/3 of that of glucose
zero-dimensional material		
Carbon quantum dots (CQD)	Fluorescent sensors in the literature show <10% signal attenuation after 28 days of storage in urine samples	Pore size sieving effect to physically block large interfering molecules
Nanodiamonds (NCD)	Sensors in the literature retain 75% of initial sensitivity after 1 month of storage	Chemically inert surfaces to reduce non-specific adsorption Surface modifications, such as boron or nitrogen doping to improve conductivity and grafting of selective membranes (e.g., polymers) to block interfering substances

### 6.1 One-dimensional materials: carbon nanotubes and nanofibers

**FIGURE 6:**
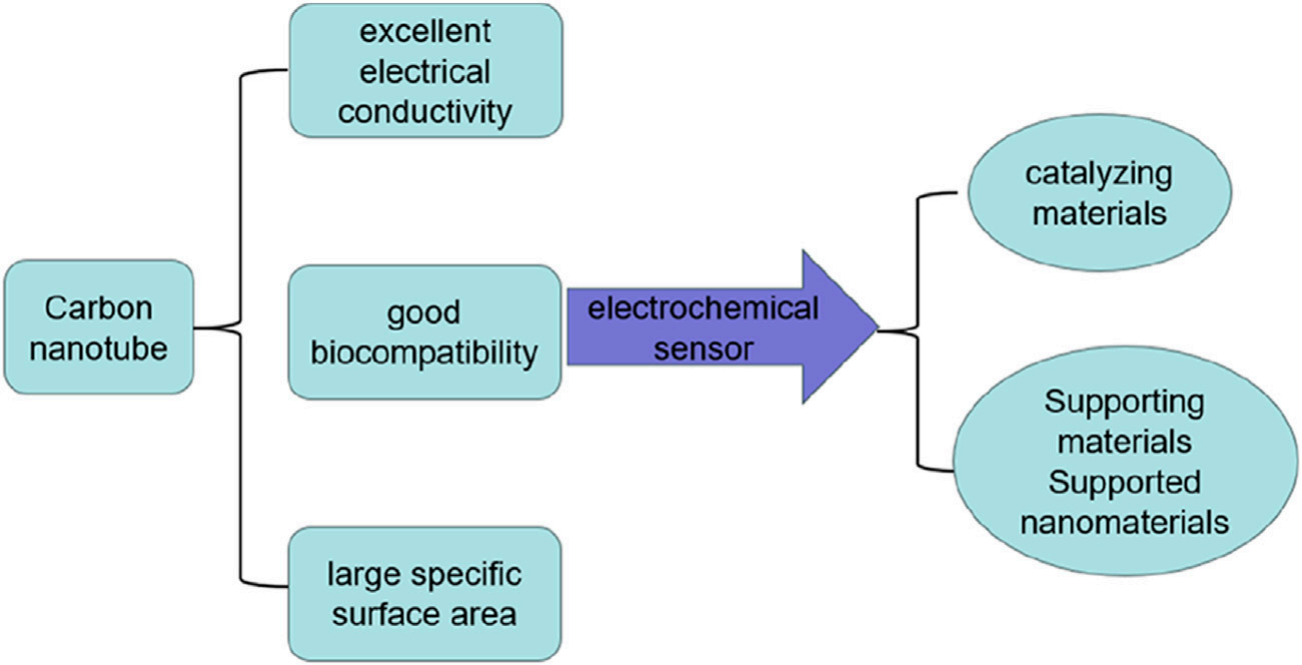
The role of carbon nanotubes in electrochemical sensors.

Carbon nanotubes are allotropes of carbon, which are part of the fullerene family of structures and are small in diameter (nanoscale) and length (micrometer) (Wang et al., 2021; Sridharan et al., 2022). Typical carbon nanotubes are tubular carbon atom systems composed of hexagonal carbon atoms, which have special properties due to their symmetrical structure. Their behavior depends entirely on their spiral nature, and because of this, they play the role of semiconductors or metals (Soni et al., 2020). Carbon nanotubes (CNTs) are highly regarded members of the synthetic carbon allotrope due to their unique arrangement of carbon atoms, sp2 hybridization, and cylindrical structure arranged between C-C distances of 1.42 Å and 3.4 Å layers, which make them different from other nanocarriers (Prajapati et al., 2022). According to their number of layers, they are divided into (a) single-walled carbon nanotubes (SWCNTs): graphite sheets of single-atom thickness bent into cylinders; (b) Multi-walled carbon nanotubes (MWCNTs): several layers of graphite sheets arranged concentrically (Bin et al., 2022). Single-walled carbon nanotubes (SWCNTs) have a simple chemical structure and clean surface properties, which give them a high degree of chemical stability. In contrast, multi-walled carbon nanotubes (MWCNTs) have a more complex wall layer, resulting in a complex and variable surface structure, which can adsorb and bind a large number of surface functional groups. This structural complexity provides significant advantages for MWCNTs in a wide range of applications (Han et al., 2024; Xia et al., 2023; Su et al., 2023) (Figure 6).

Carbon nanotubes were introduced by Sumio Iijima in 1991 (Sumio, 1991) and formally described and named, its unique tubular nanostructure (L/D ratio may be as high as 132 million:1), superior strength, and significant physicochemical properties quickly caught the attention of researchers (Upadhyay et al., 2011; Zeng et al., 2022). In addition, CNTs have excellent electrical conductivity, strong adsorption capacity, high sensitivity, good biocompatibility, and excellent chemical stability, making them ideal nanomaterials for the preparation of biosensors (Ma et al., 2019; Wang et al., 2021; Wang et al., 2009). Kyuhwan et al. discovered that carbon nanotubes (CNTs) as covalently immobilized materials for Gox can effectively maintain Gox based on activity and stability, the biosensor prepared based on this was used to detect glucose, and the sensitivity reached 53.5 μA·mM^-1^cm^-2^, which remained 86% active after 2 weeks, compared to the DTSSP modified gold electrode (0.026 s^-1^) and glassy carbon electrode (0.2 s^-1^), this electrode achieved an electron transfer rate of 1.14 s^-1^, a 5.7∼44-fold improvement (Hyun et al., 2015). Yin et al. (2024) used a glucose monitoring skin patch prepared from a hollow syringe modified with glucose oxidase (GOD) and carbon nanotubes (CNTs) as an electrochemical sensor for glucose monitoring and an integrated circuit for signal processing and transmission, and displaying real-time blood glucose levels on a smartphone via Bluetooth, which continuously measures glucose in real time in live animals with micromolar sensitivity and a lifetime of more than 14 days of useful life.

**FIGURE 7:**
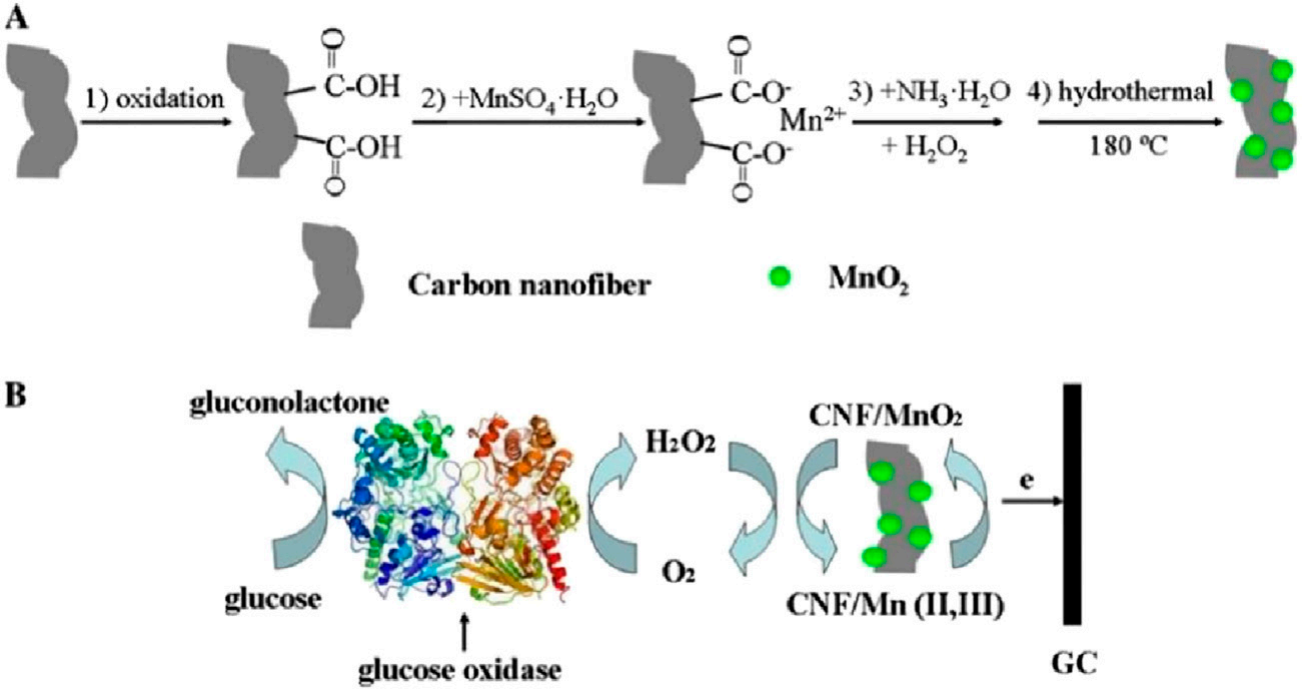
**(A)** Schematic illustration for the synthesis of MnO_2_–CNFs nanocomposites. the MnO_2_–CNFs conjugates were prepared via the interaction of the carboxyl group with Mn^2+^ for subsequent on-spot chemical deposition of MnO_2_ onto CNFs. **(B)** Schematic representation of the mechanism of electrocatalysis of glucose catalyzed by glucose oxidase. Reproduced with permission from ref, (Zhang et al., 2018b). Copyright 2018, Springer Science Publication.

Carbon nanofibers (CNFs), with their excellent electrical conductivity and remarkable specific surface area, have attracted much attention in nanotechnology and materials science in recent years. Its excellent electron mobility ensures that electrons can be transferred at the electrode interfaces in an efficient and low-resistance manner, thus realizing an efficient electrical signal transduction mechanism. In addition, the nanoscale structure of CNFs provides many active sites, further facilitating rapid electron transfer and reaction kinetics. For example, Zhang et al. (2018a) modified GOx electrodes by combining manganese dioxide nanoparticles and carbon nanofiber nanocomposites and completed practical application validation by spiking samples of urine in order to obtain sensors with detection limits as low as 0.015 mM, recoveries in the range of 101.0%–104.8%, and retention of 71.9% of activity after 30 days, and the sensitivity of the MnO_2_- CNFs-modified sensor was 5.4 times more sensitive to H_2_O_2_ (33.1 μA/mM) than the MnO_2_-modified electrode only (6.1 μA/mM) (Figure 7).

### 6.2 Two-dimensional materials: graphene, graphene oxide and graphyne

**FIGURE 8:**
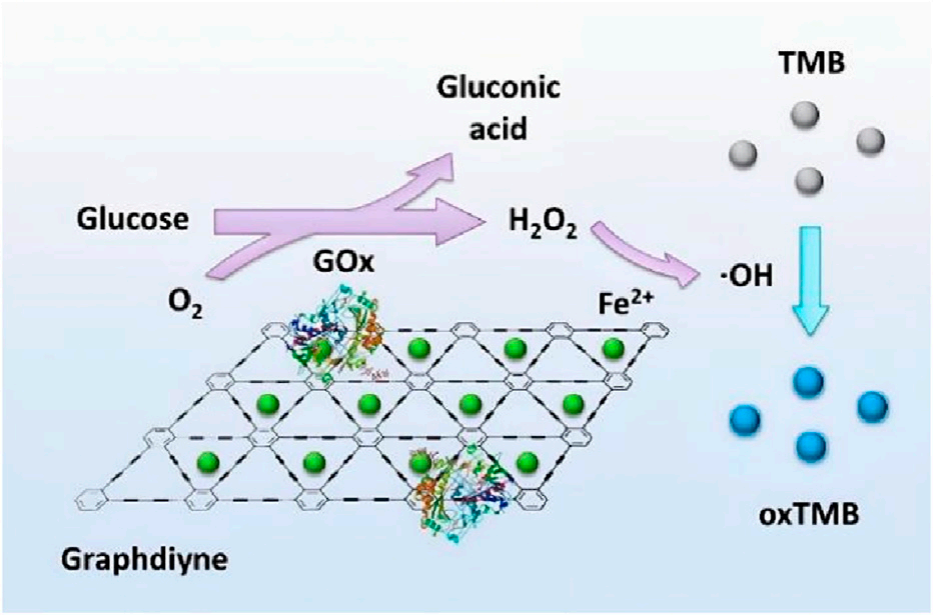
Schematic representation of the mechanism for immobilizing ferrous ions and glucose oxidase on Graphdiyne. Reproduced with permission from ref, (Liu et al., 2019). Copyright 2018, ACS Publication.

Following CNTs, scientists Geim and Novoselov prepared a new carbon nanomaterial, graphene (GR) in 2004 (Morales-Narvaez et al., 2017; Sun and Joshi, 2010; Wu et al., 2017). Graphene biosensors have been vigorously developed in the past decade due to their small size, unique conductive and optical properties (such as fluorescence quenching and conductivity), and good biocompatibility that meet the high efficiency and diversity requirements of biosensors (Zhu et al., 2017; Zhou et al., 2016; Zhao et al., 2022). Pakapongpan and Poo-Arporn, (2017); Wu et al. (2017) prepared a GOx biosensor by self-assembling glucose oxidase (GOx) on covalently modified magnetic nanoparticles (Fe_3_O_4_ NPs). The graphene material facilitated the electron transfer between the enzyme and the electrode surface, and the biosensor showed a fast amperometric response to glucose (3 s), a linear range from 0.05 to 1 mM, a low detection limit of 0.1 μM, significantly lower than that of GNs/ZnO/SPE (70 μM), and high sensitivity (5.9 μA mM^-1^), and the current response of the sensor remained 95.6% of the initial value after 1 month’s storage at 4°C. Wu et al. (2022) developed a microelectrode glucose biosensor based on 3D hybridized nanoporous platinum/graphene oxide nanostructures for rapid glucose detection in tomato and cucumber fruits, which achieved high glucose detection sensitivity (11.64 muA calibrated in glucose standard solution), low detection limit (13 mumol/L) and fast response time (95% steady-state response within 3 s). Liu et al. (2019) immobilized ferrous ions and glucose oxidase on GDY sheets and presented GDY-based composites with dual enzyme activity. Rat serum was used as a test sample and the electrodes obtained were superior to V_2_O_5_ nanowires (10–2000 µM) and Cu-Ag/GO composites (1–30 µM), with R^2^ = 0.998, and 0.89 µM for Fe-GDY/GOx, which is significantly lower than Fe/CeO_2_ NPs (3.41 µM) and H_2_TCPP/Fe_2_O_3_ NPs (2.54 µM) (Figure 8). Antonova et al. (2024) developed a soft microfluidic glucose sensor catalyzed and mediated by bimetallic palladium and platinum supported on reduced graphene oxide with 1,10-phenanthroline-5,6-dione. The sensor demonstrated a linear amperometric response to glucose within the range of 50–900 μM at an applied potential of 0.2 V, exhibiting a detection limit of 37 μM and a sensitivity of 30 μA cm^-2^ mM^-1^. The wearable sensor prototype enables convenient non-invasive measurement of exercise-induced sweat glucose levels for personalized diabetes monitoring.

### 6.3 Three-dimensional materials: hollow carbon spheres and carbon aerogels

Carbon aerogels (CAs) are mesoporous materials with abundant porosity and high specific surface area that are suitable for many practical applications. In addition, CAs have been found to have excellent biocompatibility due to their three-dimensional (3D) structural network, good electrical conductivity, exceptional chemical and environmental stability, and strong adhesion capabilities, which are ideal for the development of next-generation catalyst materials (Thirumalraj et al., 2018). Yu et al. (2015) reported a zirconium phosphate-carbon aerogel (ZrP-CA) composite material, where the ZrP-CA/GOx configuration exhibited a linear calibration range of 0.12–2.0 mM for glucose detection, surpassing conventional GOD/In_2_O_3_–chitosan (0.005–1.3 mM) and GOD–graphene–CdS (0.025–1.19 mM) systems. The sensitivity reached 5.56 μA mM^-1^cm^-2^, demonstrating a enhancement compared to traditional ZrP-based sensors (0.41 μA mM^-1^cm^-2^).

### 6.4 Zero-dimensional materials: carbon quantum dots and nanodiamonds

Carbon quantum dots (CDs) are a class of fluorescent carbon-based nanoparticles with a particle size of <10 nm, and the abundant oxygen-containing functional groups (-OH, -COOH) on their surfaces provide multiple anchor sites for Gox immobilization. Compared with conventional materials, the advantages of CDs are reflected in the excitation wavelength-dependent fluorescence property of CDs that can work synergistically with electrochemical signals, and the cytotoxicity of CDs (IC_50_ > 500 μg/mL) is significantly lower than that of CNTs (IC_50_ ≈ 50 μg/mL) for implantable sensors. Hu et al. (Hu et al., 2020) developed a sensitive fluorescent microfluidic sensor based on carbon quantum dots (CQDs), cadmium telluride quantum dots (CdTe QDs) aerogel, and glucose oxidase (GOx), with all experimental validations performed using human urine specimens for glucose detection. The sensor demonstrated exceptional storage stability, retaining stable fluorescence signal (R/G ratio) and colorimetric response after 30-day storage at −20°C. It achieved a broad glucose detection range from 0 to 13 mM with a detection limit of 0.223 mM (S/N = 3). Zhao et al. (2006) developed an electrochemically pretreated glucose biosensor based on non-doped nanocrystalline diamond (N-NCD)-modified gold electrodes for selective glucose detection, achieving a broad linear calibration range from 10 μM to 15 mM with a low detection limit of 5 μM (S/N = 3), which significantly outperforms conventional sensors. The biosensor retains 75% of its initial sensitivity after 30 days of storage.

We compare the effects of different carbon nanomaterials on glucose sensors (Table 7) and add the mechanisms by which they increase the electron transfer rate of the sensors (Table 8).

**TABLE 7:** Comparison of different carbon nanomaterials for glucose sensor applications.

Material	Core advantage	Sensitivity	Selectivity	Stability	Cost
Carbon nanotubes	High electrical conductivity, mechanical strength, wide detection range	High	Medium	Medium	Medium
Graphene	Ultra-high electron transfer rate, large specific surface area	Very high	High	High	High
Graphene oxide	Easy surface modification, synergistic effects	High	Extremely high	High	Medium
Carbon quantum dots	Fluorescence properties, low detection limit	Extremely high	High	High	Low
Carbon aerogel	3D porous structure, high loading capacity	Medium	Medium	Medium	High
Nanodiamond	Chemical stability, biocompatibility	Medium	High	Extremely high	Extremely high
Graphdiyne	High electrical conductivity and unique pore structure	Extremely high	Extremely high	Medium	High
Carbon nanofibers	3D network structure, easy surface functionalization	Medium	Medium	High	Medium

**TABLE 8:** Effect of different carbon nanomaterials on the electron transfer rate of glucose sensors.

Material	Influence mechanism		Main limitations
Carbon nanotubes	One-dimensional conductive channels: π-π conjugation effect of tubular structure provides direct electron transfer pathway, lower interfacial resistance	High aspect ratio: enhanced contact area with enzymes, promotes direct electron transfer (DET) for glucose oxidase (GOx)	Poor dispersibility, potential biotoxicity
Graphene	Ultra-high electrical conductivity: 2D honeycomb structure of sp^2^ hybridized carbon atoms creates continuous electron channels, high electron mobility, significantly shortens electron transfer path	Large specific surface area (2,630 m^2^/g): exposes more active sites, enhances loading efficiency of enzymes or catalysts, promotes interfacial charge transfer	Reduced number of active sites due to interlayer stacking
Graphene oxide	Surface functional groups: oxygen-containing groups (-OH, -COOH) enhance immobilization of biomolecules (e.g., enzymes) but block conductivity	Reduction treatment (rGO): restoration of part of the sp^2^ structure by thermal or chemical reduction, significant increase in electrical conductivity (close to 80% of graphene)	Reduction treatment is required to restore conductivity
Carbon aerogel	3D porous network: high porosity (>90%) shortens ion diffusion paths, facilitates electrolyte penetration, and reduces charge transfer impedance	Conductive backbone: graphene or carbon nanotubes reinforced 3D structure provides continuous electron transport paths	Complex preparation process
Carbon quantum dots	Quantum size effect: small size (<10 nm) provides high surface activity but low conductivity, need to be compounded with conductive substrates (e.g., GO, CNTs)	Fluorescence-electrochemical synergy: enhanced catalytic efficiency through light-induced electron transfer	Need to compound with conductive substrate to compensate for conductivity

## 7 Synergistic chemical modification of carbon nanomaterials and enzymes with glucose sensors

The high conductivity of carbon nanomaterials provides a fast channel for electron transfer between the enzyme and the electrode; their large specific surface area also provides abundant sites for enzyme immobilization, while chemical modification can shorten the electron transfer path and further reduce the interfacial resistance; by regulating the enzyme microenvironment, conformational changes can be reduced, which improves the catalytic efficacy and thermal stability of the enzyme. The adsorption properties of carbon nanomaterials synergize with the selective screen of chemical modification, which can effectively shield interferences such as ascorbic acid and uric acid and improve the specificity of the sensor (Table 9).

**TABLE 9:** Comparison of typical cases of synergies.

Carbon nanomaterials	Enzyme modification strategies	Performance enhancement	References
Graphene	Covalent modification of Gox by pyrene moiety	Extended detection range to 0.01–50 mM, 3-fold increase in sensitivity	Liu et al. (2013)
Multi-walled carbon nanotubes	Covalent immobilization of Gox by amino groups	Sensitivity 53.5 μA-mM-^1^-cm-^2^, 2-week activity retention 86%	Hyun et al. (2015)
Carbon quantum dots	Electrostatic adsorption of Gox	Detection limit 0.223 mM, significant biocompatibility	Hu et al. (2020)
Graphyne	Fe^2+^ co-loading with Gox	Linear range 5–160 μM, R^2^ = 0.998	Liu et al. (2019)

## 8 Significance and challenges of glucose oxidase electrode modification

Glucose oxidase electrochemical sensors have attracted much attention because of their ability to detect glucose specifically, but maintaining the activity of the enzyme is also a challenge. By introducing materials such as carbon nanotubes, graphene, and metal nanoparticles with high specific surface area, good electrical conductivity, and catalytic activity on the electrode surface and by chemically modifying the enzyme, the efficiency of electron transfer between the enzyme and the electrode can be significantly improved, thereby increasing the sensitivity of the sensor. These modified materials not only provide more attachment sites for GOx, but also enhance the interaction between the enzyme and the electrode, making the oxidation of glucose more efficient. For sensor performance, future research should focus on optimizing sensor sensitivity, improving selectivity and interference immunity, and increasing long-term stability and commercialization potential.

In recent years, some enzyme-modified nanomaterial sensors have entered the commercialization stage, e.g., FreeStyle Libre (Abbott) has successfully occupied a significant position in the global market of glucose detection. However, the following key issues still need to be addressed in order to realize their large-scale production: reproducibility of material synthesis (e.g., the diameter and chirality control of single-walled carbon nanotubes (SWCNTs) still relies on a complex gas-phase deposition process, resulting in large performance variations between batches), compatibility of enzyme immobilization processes (existing enzyme modification technologies (e.g., covalent modification, macromolecular coupling) are susceptible to environmental fluctuations in continuous production), device miniaturization and integration (lab lab labs are susceptible to environmental fluctuations), and the use of enzyme-enabled devices.), miniaturization and integration of equipment (laboratory sensors rely on bulky electrochemical workstations, while commercial equipment requires integrated signal processing modules).

## 9 Outlook for future applications of the glucose oxidase electrode

Most of the current research is still based on standard solutions or simulated samples, and the validation of real blood samples needs to be further optimized for immunity (e.g., ascorbic acid, uric acid, etc.) and long-term stability. Future studies need to focus on the validation of sensor performance in real complex samples (e.g., whole blood, food extracts) and the development of portable devices in combination with miniaturization techniques to meet the needs of clinical diagnosis and immediate testing (POCT). At the same time, there are many issues to be faced to realize the utility of glucose oxidase electrodes as follows. Existing carbon nanomaterials (e.g., graphene, carbon nanotubes) may trigger an inflammatory response upon long-term skin contact, so encapsulating materials that are flexible, breathable, and biologically inert, such as polyurethane-nanofibrillar cellulose composite membranes, need to be developed. At the same time, surface functionalization techniques (e.g., PEG modification) are used to reduce the immunogenicity of the materials, thus further improving their biocompatibility. Second, wearable devices need to adapt to sweat pH fluctuations, mechanical deformation and temperature changes to ensure sensor stability in dynamic environments. Meanwhile, continuous glucose monitoring requires the integration of multiple sensors (e.g., pH, temperature compensation modules), but the energy consumption of nanomaterial devices limits miniaturization, so multimodal data synchronization needs to be optimized to balance energy consumption.
